# Clinical variables predicting the risk of a hospital stay for longer than 7 days in patients with severe acute exacerbations of chronic obstructive pulmonary disease: a prospective study

**DOI:** 10.1186/s12931-018-0951-4

**Published:** 2018-12-27

**Authors:** Ernesto Crisafulli, Antonella Ielpo, Enric Barbeta, Adrian Ceccato, Arturo Huerta, Albert Gabarrús, Néstor Soler, Alfredo Chetta, Antoni Torres

**Affiliations:** 1 Department of Medicine and Surgery, Respiratory Disease and Lung Function Unit, University of Parma, Parma, Italy; 2 Pneumology Department, Clinic Institute of Thorax (ICT), Hospital Clinic of Barcelona, Institut d’Investigacions Biomèdiques August Pi i Sunyer (IDIBAPS), University of Barcelona, Ciber de Enfermedades Respiratorias (CIBERES), Villarroel 170, 08036 Barcelona, Spain; 3 Icrea Academia, Barcelona, Spain

**Keywords:** Chronic obstructive pulmonary disease, Acute exacerbation, Hospital stay, Dyspnoea, acute respiratory acidosis

## Abstract

**Background:**

Chronic obstructive pulmonary disease (COPD) patients may experience an acute exacerbation (AECOPD) that requires hospitalisation. The length of hospital stay (LHS) has a great economic impact on the health-care system. Knowing the predictors of prolonged LHS could help to identify possible interventions.

**Methods:**

We performed a prospective study to identify the clinical predictors of prolonged LHS in patients hospitalised for AECOPD. We divided the study sample by LHS into normal (≤7 days) and prolonged LHS (> 7 days) groups. Outcomes were the need for non-invasive and invasive mechanical ventilation (NIMV and IMV), intensive care unit (ICU) admission, and the 3-year mortality.

**Results:**

We enrolled 437 patients, of which 213 and 224 had normal LHS and prolonged LHS, respectively. Patients with a prolonged LHS had more prior hospitalisations for AECOPD, a worse mMRC (modified Medical Research Council) dyspnoea score, a higher prevalence of long-term oxygen therapy and a higher rate of congestive heart disease. During the current admission, this group also tended to require NIMV, IMV and ICU admission and the mortality risks at 6 months, 1 year and 3 years were higher. In the multivariate regression analysis, an mMRC dyspnoea score ≥ 2 (odds ratio-OR 2.24; 95% confidence interval-CI 1.34 to 3.74; *p* = 0.002) and the presence of acute respiratory acidosis (OR 2.75; 95% CI 1.49 to 5.05; *p* = 0.001) predicted a prolonged LHS at admission.

**Conclusions:**

The presence of an mMRC ≥2 and acute respiratory acidosis at admission independently increased the risk of a prolonged LHS for AECOPD.

## Introduction

The recent Global Burden of Disease study reported that chronic obstructive pulmonary disease (COPD) was a prevalent cause of death worldwide [1]. Acute exacerbations of COPD (AECOPD), which are characterised by a worsening of respiratory signs and symptoms and an increasing of domiciliary therapies [2], may occur during the clinical course of disease and increase the mortality. Related to this aspect, some patients need hospitalisation depending on the severity of the AECOPD [2, 3] and if it prolonged, this can increase the costs of managing the disease [4].

The length of hospital stay (LHS) for an AECOPD is related to several factors. These include age [5], disease severity [5, 6], the presence of comorbidities [7, 8], high carbon dioxide partial pressures (PCO_2_) [7], the need of mechanical ventilation [6] or an intensive care unit (ICU) [9], a low serum albumin level [7], the dyspnoea perception [10] and the respiratory rate [8]. Other variables, such as admissions at the weekend [8] and social factors [11], have also been considered relevant. However, three aspects need to be highlighted in these studies. First, some of them were based on audit data [6], medical records [7] or retrospective analysis [10, 11] and not from prospective observational data. Second, different thresholds have been used to define a prolonged LHS (e.g., 4 days [8], 8 days [10], 9 days [9], or 11 days [7]), despite evidences that the normal LHS required for AECOPD should be 6 or 7 days [12], based on the occurrence and timing of complications. Third, no studies have evaluated several clinical variables all together at the time and before hospital admission.

In this prospective study, we aimed to identify the clinical variables at the time and before hospital admission that increase the risk of prolonged LHS (defined as > 7 days) in patients hospitalised for AECOPD. Due to the impact of LHS on clinical, social and economic outcomes, we believe that identifying the predictors of prolonged LHS could help clinicians to develop targeted interventions for these patients.

## Methods

### Study cohort

We performed a prospective study at the Hospital Clinic of Barcelona, Spain, over a 7-years period from May 2009 to May 2016. We systematically enrolled all patients admitted for an AECOPD to our pneumology department. Included patients had to meet the diagnostic criteria for COPD, as set out by the Global Initiative for Chronic Obstructive Lung Disease (GOLD) [2]. For the diagnosis, we considered spirometry measurements in the stable phase at least six months before hospital admission and patients were considered positive for smoking if they had a history of 20 pack-years [2]. According to the GOLD document [2], AECOPD was defined as a recent worsening of respiratory symptoms that required a change in domiciliary therapy, with the need for hospitalisation based on the severity of AECOPD and the presence of indicators [2]. According to the literature [12] and related to the median value of the LHS in our study sample that was 7 days, patients with an AECOPD were divided into a group with normal LHS (≤7 days) and a group with prolonged LHS (> 7 days). We excluded patients with a documented history of other concomitant chronic respiratory diseases (e.g., asthma, cystic fibrosis or interstitial lung disease) or those in whom a community-acquired pneumonia or acute heart failure were identified by clinical signs, chest X-ray or computed tomography at admission.

The hospital’s ethics committee approved the study protocol (CEIC 2008/4106), and we conducted the study according to the requirements of Good Clinical Practice and the declaration of Helsinki, including later revisions. All participants gave signed informed consent.

### Microbiological sample collection

On the first day of hospitalisation, we collected sputum from a spontaneous cough sample; if adequate (a count of > 25 leukocytes and < 10 epithelial cells per field) it was processed by Gram staining and culture. In patients who did not provide a spontaneous sputum sample, we obtained an induced sputum production by inhalation of a 5% hypertonic saline solution delivered via nebuliser for 5 to 10 min.

### General measurements

We recorded data about demographic variables, body mass index, smoking habit (current or former, including the number of pack-years), number of comorbidities (using the Charlson index), prevalence of ischaemic heart disease and diabetes, dyspnoea grade (measured by the modified Medical Research Council [mMRC] scale), disease severity (using the COPD severity score [COPD-SS] questionnaire) and use of long-term oxygen therapy (LTOT). We also collected data about the season of occurrence of AECOPD, the characteristics and numbers of AECOPD events in the last year, and details of home care medications (short-acting β_2_ agonists, long-acting β_2_ agonists, anticholinergics and inhaled corticosteroids).

We recorded body temperature, respiratory rate, heart rate and blood pressure (systolic and diastolic) at admission. In addition, we recorded gas analysis (pH, partial arterial carbon dioxide pressure [PaCO_2_], the ratio of partial arterial oxygen pressure to the fraction of inspired oxygen [PaO_2_/FiO_2_], serum bicarbonate [HCO_3_^−^], and base excess [BE]), systemic response (i.e., leukocytes, haematocrit, haemoglobin, C-reactive protein [CRP], glucose and creatinine) as both at admission and at day 3. Finally, we recorded how many patients used systemic corticosteroids and antibiotics, the antibiotic classes and the duration of antibiotic treatment.

### Outcomes

Clinical progression was evaluated based on the need for non-invasive and invasive mechanical ventilation (NIMV and IMV, respectively) and for admission to ICU. Data on prognosis (i.e., cumulative number of all-cause deaths and estimated time to death) were recorded during follow-up at 30 days, 6 months, 1 year and 3 years. The date of death was identified using centralised registries.

### Statistical analysis

We reported categorical variables as numbers and percentages and we reported continuous variables as means ± standard deviations or as medians (1st quartile; 3rd quartile) for normal and non-normal distributions, respectively. Categorical variables were compared using the *X* [2] test or the Fisher exact test, while continuous variables with the *t* test or the non-parametric Mann-Whitney test, as appropriate. All statistical analyses were performed using IBM SPSS, version 25.0 (IBM Corp., Armonk, NY, USA). A *p*-value of < 0.05 was considered statistically significant.

Univariate and multivariate regression logistic models were performed with the stepwise method to predict the probability of a prolonged LHS (the dependent variable). The independent variables in the univariate analyses were as follows: mMRC dyspnoea score (≥ 2); GOLD 2017 stages A, B, C or D; number of previous AECOPD episodes requiring hospitalisation (≥1); use of LTOT; symptom duration before admission (≥7 days); pre-admission therapy, including antibiotic use within 3 months, salbutamol use within two weeks and ipratropium bromide use within two weeks; the COPD-SS (≥15); the presence of a comorbidity such as ischaemic or congestive heart disease, diabetes or chronic kidney failure); the presence of acute respiratory acidosis (pH < 7.35), at admission and day 3, hypercapnia (PCO_2_ > 45 mmHg), acute severe hypoxemia (PaO_2_/FiO_2_ ratio < 200) and renal bicarbonate retention (HCO_3_ > 30 mmol/L); NIMV use; a positive sputum culture for *Pseudomonas aeruginosa* in the previous year or during hospitalisation, a microorganism resistant to conventional treatment (MRCT) or *Streptococcus pneumoniae*; antibiotic use during hospitalisation. Variables that showed a significant result (*p* < 0.1) were included in the subsequent multivariate regression stepwise model. To detect collinearity, we calculated the *r* coefficient of two variables and for those highly correlated (*r* > | ± 0.30|) the variable with the largest variance was excluded from the multivariate analysis [13]. Odds ratios (ORs) and 95% confidence intervals (CI) were then calculated.

Time-to-event variables were analysed by Kaplan–Meier survival curves and the Gehan–Breslow–Wilcoxon test was applied to emphasise early differences [14]. Patients lost to follow-up were censored in the survival analysis. Cox proportional hazard regression models were used to report the mortality at 30 days, 6 months, 1 year and 3 years [15], and we calculated the hazard ratios and 95% CIs.

## Results

### General data on study cohort

**Fig. 1:**
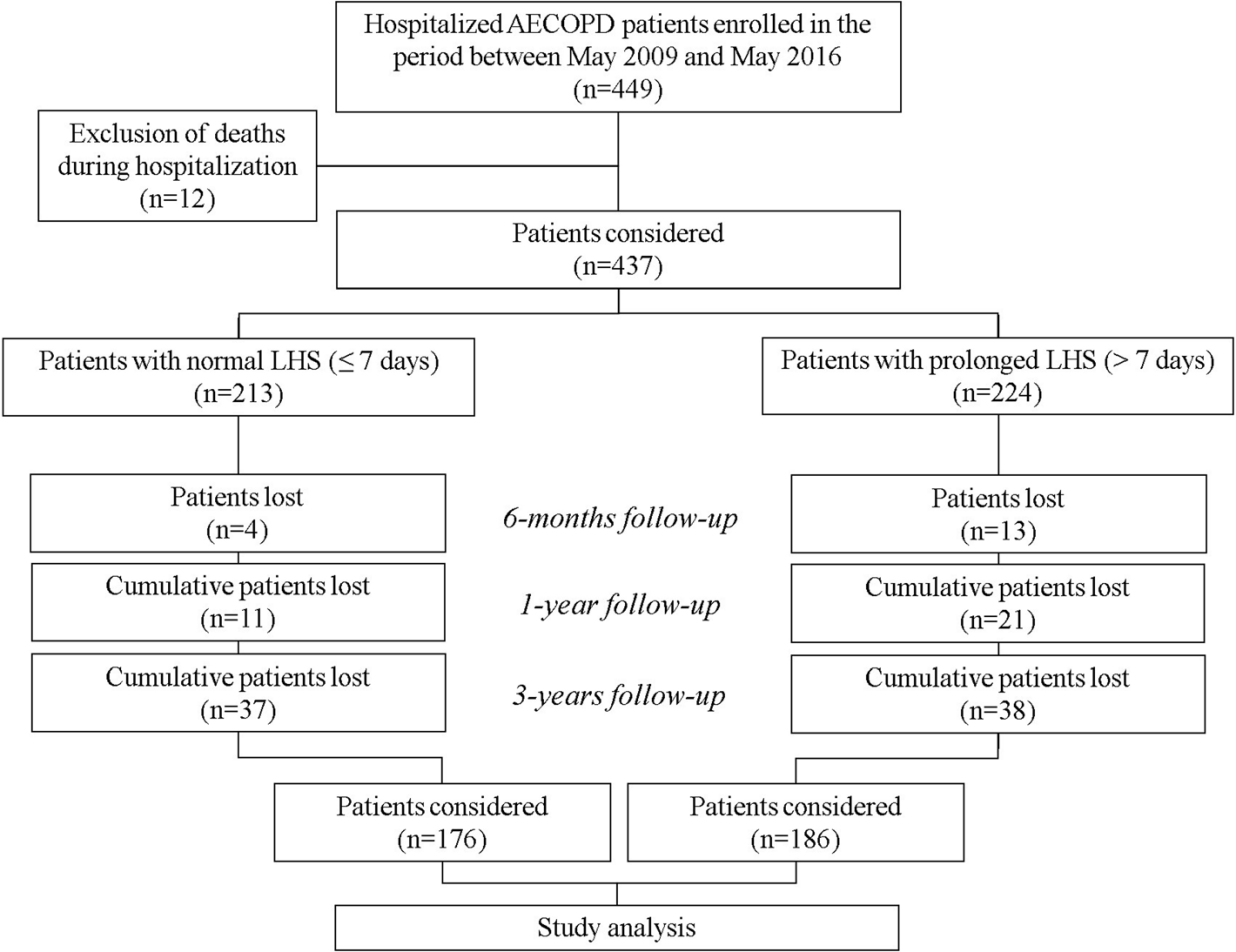
Study flow chart

Over the 7-year study period, we enrolled 449 patients admitted for an AECOPD; of these, 12 died during hospitalisation and so excluded. Among the 437 remaining patients, 213 had a normal LHS and 224 had a prolonged LHS. In comparison to the normal LHS group, the prolonged LHS group had higher rates of GOLD D stage, LTOT, associated congestive heart disease and prior hospitalisations for AECOPD. The prolonged LHS group had also worse dyspnoea perception, longer symptom duration before admission and greater requirement of salbutamol and ipratropium in the two weeks before admission. Figure 1 shows the flow diagram for the study and Table 1 shows the baseline characteristics.

**Table 1:** Baseline characteristics of patients by the length of hospital stay

Variables	Normal LHS (≤ 7 days) *(n = 213)*	Prolonged LHS (> 7 days) *(n = 224)*	*p* value
*Domiciliary medications*			
Age, years	73 [64.5; 78]	72 [64; 78]	0.857
Male, %	82	80	0.637
BMI, kg/m^2^	27 [24.2; 29.4]	27.6 [23.4; 31.5]	0.486
Smoking habit: Current/Former, %	41/59	39/61	0.598
Pack/year	50 [40; 80]	60 [40; 80]	0.797
FEV_1_, % predicted	44 [33; 63]	42 [30; 58]	0.126
FEV_1_/FVC	49.5 [38.7; 62]	49 [36; 62.7]	0.823
GOLD 2017 stages: A/B/C/D, %	32/32/15/21	18/39/12/31	**0.015**
LTOT, %	22	32	**0.013**
mMRC dyspnoea grade	2 [1; 3]	2 [1; 4]	**0.001**
COPD-SS severity questionnaire	13 [8; 18]	14 [9; 19]	0.108
Charlson index	2 [1; 3]	2 [1; 3]	0.750
Ischaemic heart disease, %	11	9	0.501
Congestive heart disease, %	9	18	**0.008**
Diabetes, %	21	23	0.618
Chronic kidney failure, %	4	7	0.257
Season of admission: Winter/Spring/Summer/Autumn, %	40/14/29/17	45/16/25/14	0.508
Period of admission: years 2009–2011/2012–2104/2015–2017, %	61/31/8	58/30/12	0.463
Previous AECOPD^a^	0 [0; 1]	0 [0; 2]	0.286
Patients with ≥2 previous AECOPD^a^, %	24	27	0.501
Previous AECOPD requiring hospitalisation^a^	0 [0; 1]	0 [0; 1]	**0.030**
Patients with ≥1 previous AECOPD requiring hospitalisation^a^, %	28	37	**0.029**
Onset of symptoms until admission, days	4 [2; 7]	5 [3; 7.75]	**0.035**
*Use of antibiotics previous admission*			
In a period of one week before, %	24	23	0.924
Days of treatment	7 [3; 10]	6 [3; 7]	0.625
Penicillins/Fluoroquinolones/Macrolides/Cephalosporins/Others, %	30/48/11/2/9	24/40/18/11/7	0.248
In a period of three months before, %	40	53	**0.044**
Penicillins/Fluoroquinolones/Macrolides/Cephalosporins/Others, %	23/63/5/0/9	34/45/10/8/3	0.149
*Use of other drugs in a period of two weeks before admission*			
Systemic corticosteroids, %	12	13	0.867
Salbutamol, %	4	14	**0.001**
Ipratropium bromide, %	5	13	**0.005**
*Domiciliary medications*			
Salbutamol only, %	3	3	0.922
Anticholinergic only, %	5	5	0.819
LABA + Anticholinergic, %	2	1	> 0.999
LABA + ICS, %	3	2	> 0.999
Anticholinergic + ICS, %	2	1	> 0.999
LABA + Anticholinergic + ICS, %	36	37	0.828

Data are shown as number of patients (percentage) or medians [1st quartile; 3rd quartile], unless otherwise stated. Percentages are calculated for non-missing data

LABAs include salmeterol, formoterol and indacaterol; Anticholinergics include ipratropium and tiotropium; and ICS include budesonide and fluticasone

^a^Previous AECOPD were considered if occurring in a period of the preceding year

*Abbreviations: BMI* indicates body mass index; *COPD*, chronic obstructive pulmonary disease; *COPD-SS*, COPD severity score questionnaire; *FEV*_*1*_, forced expiratory volume in the 1st second; *FVC*, forced vital capacity; *GOLD*, Global Initiative for Chronic Obstructive Lung Disease; *ICS*, inhaled corticosteroids; *LABA*, long-acting β_2_ agonist; *LHS*, length of hospital stay; *LTOT*, long-term oxygen therapy; *mMRC*, modified Medical Research Council

### Clinical, laboratory and microbiological variables

**Fig. 2:**
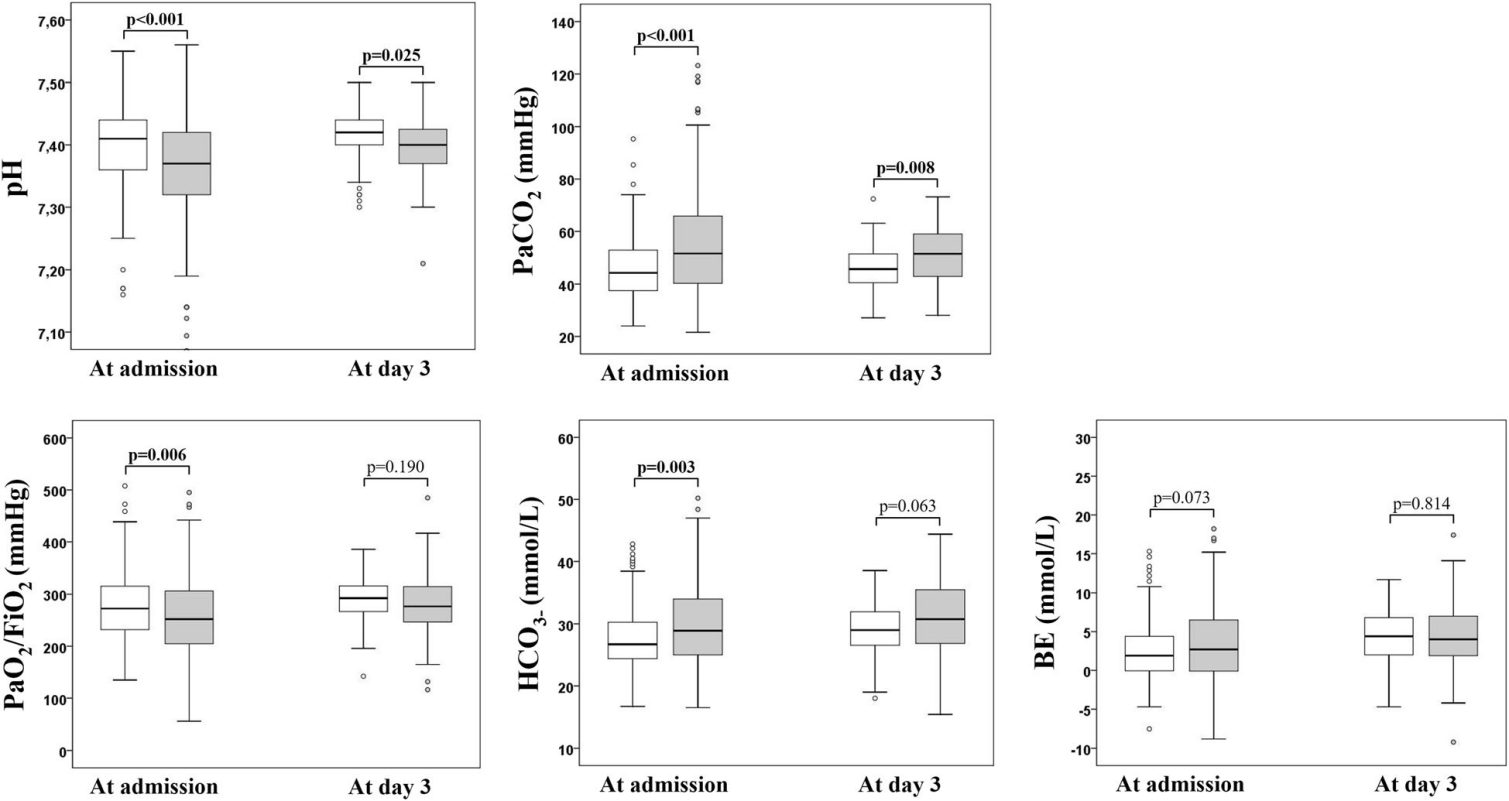
Blood gas analysis on admission and day 3. White and grey represent the AECOPD patients with normal LHS and prolonged LHS, respectively. *Abbreviations:* PaCO_2_, partial pressure of arterial carbon dioxide; PaO_2_/FiO_2_, ratio of partial pressure of arterial oxygen to the fraction of inspired oxygen; HCO_3_^−^, serum bicarbonate; BE, base excess

Blood gas analysis at admission (Fig. 2) revealed worse values in the prolonged LHS group than in the normal LHS groups for pH (median [1st quartile; 3rd quartile] 7.37 [7.32; 7.42] versus 7.41 [7.36; 7.44]), PaCO_2_ (51.7 mmHg [40.3; 66] versus 44.3 mmHg [37.4; 53]), PaO_2_/FiO_2_ (251.9 [204.2; 306.6] versus 272.3 [231.6; 315.7]), HCO_3−_ (28.9 mmol/L [24.9; 34] versus 26.7 mmol/L [24.4; 30.3]). At day 3, different levels for pH and PaCO_2_ were confirmed in the study groups. In the prolonged LHS group, fluoroquinolone use was lower and cephalosporin use was higher when compared with the normal LHS group. All other clinical and laboratory variables were similar between the study groups (Table 2).

**Table 2:** Clinical and laboratory variables

Variables	Normal LHS (≤ 7 days) *(n = 213)*	Prolonged LHS (> 7 days) *(n = 224)*	*p* value
Respiratory rate, b/min	24 [20; 28]	24 [20; 28]	0.482
Heart rate, b/min	91.5 [83; 102.5]	95 [80; 108]	0.293
Body temperature, °C	36.4 [35.9; 37]	36.3 [36; 36.8]	0.569
SBP, mmHg	140 [120; 155]	135.5 [124; 156]	0.719
DBP, mmHg	75 [68; 86]	76 [68; 87]	0.895
*Laboratory variables at admission*			
Leucocytes, 10^9^/l	10.1 [7.9; 14]	10.1 [7.7; 13.6]	0.464
Haematocrit, %	43 [39; 47]	43 [40; 47]	0.425
Haemoglobin, g/L	138 [125; 151]	140 [127; 153]	0.738
C-reactive protein, mg/dL	4.1 [1.3; 10.1]	3.6 [1.1; 9.7]	0.412
Glucose, mg/dL	123 [108; 152.7]	126 [107.7; 166]	0.485
Creatinine, mg/dL	0.9 [0.8; 1.1]	0.9 [0.7; 1.1]	**0.041**
*Laboratory variables at day 3*			
Leucocytes, 10^9^/l	10.6 [8.3; 13.4]	10.7 [8.1; 13]	0.922
Haematocrit, %	41 [38; 44]	41 [37; 45]	0.685
Haemoglobin, g/L	131 [119; 143]	131 [118; 143]	0.436
C-reactive protein, mg/dL	1.4 [0.5; 4.1]	0.9 [0.3; 2.9]	0.161
Glucose, mg/dL	116 [96; 153]	123 [100.5; 161]	0.096
Creatinine, mg/dL	0.9 [0.7; 1.2]	0.8 [0.7; 1]	**0.027**
*Treatments during hospitalisation*			
Systemic corticosteroids, %	91	92	0.631
Antibiotics, %	84	88	0.304
Number of antibiotics used, 0/1/≥2, %	16/64/20	12/64/24	0.383
Duration of antibiotic treatment, days	7 [5; 10]	7 [6; 10]	0.375
Penicillins, %	16	19	0.498
Fluoroquinolones, %	57	45	**0.023**
Macrolides, %	2	2	> 0.999
Cephalosporins, %	1	5	**0.035**
Carbapenems, %	0	1	0.501

Data are shown as number of patients (percentage) or medians [1st quartile; 3rd quartile], unless otherwise stated. Percentages are calculated for non-missing data

Systemic corticosteroids include methylprednisolone; penicillins include amoxicillin and amoxicillin/clavulanate; fluoroquinolones include ciprofloxacin, moxifloxacin and levofloxacin; macrolides include azithromycin and clarithromycin; cephalosporins include ceftriaxone, cefotaxime, cefuroxime and cefepime; and carbapenems include meropenem

*Abbreviations: DBP* and *SBP* indicate diastolic and systolic blood pressure, respectively; *LHS*, length of hospital stay

Table 3 summarises the results of microbiological testing. In the year before hospitalisation, the prevalence of patients with positive cultures, ≥1 pathogens or positive samples for *P. aeruginosa* or MRCT was higher in the prolonged LHS group compared with the normal LHS group. In the samples collected during hospitalisation, *P. aeruginosa* and MRCT occurred more frequently in the prolonged LHS group, while *S. pneumoniae* occurred less frequently. Finally, the prevalence rates of *P. aeruginosa* and MRCT colonisation were higher in the prolonged LHS group (100 and 90% of cases, respectively).

**Table 3:** Microbiological variables

	Sputum sample collected in the previous year the hospitalisation				Sputum sample collectedduring the hospitalisation			
	Number of cases	Normal LHS^a^ (≤ 7 days)	Prolonged LHS^a^ (> 7 days)	*p* value	Number of cases	Normal LHS^a^ (≤ 7 days)	Prolonged LHS^a^ (> 7 days)	*p* value
Patients with positive cultures	63	33	67	**0.008**	91	35	43	0.179
Number of pathogens: 0	373	51	49	**0.031**	346	51	49	0.138
1	43	33	67		89	39	61	
≥ 2	20	35	65		2	50	50	
*Pseudomonas aeruginosa*	16	13	87	**0.041**	25	12	88	**0.001**
*Pseudomonas aeruginosa* colonisation^c^	–	–	–	–	9	0	100	**0.002**
*Haemophilus influenzae*	5	60	40	0.323	16	31	69	0.454
*Streptococcus pneumoniae*	7	43	57	0.677	18	61	39	**0.037**
*Streptococcus spp.*	5	20	80	0.657	–	–	–	–
*Staphylococcus spp.*	4	50	50	0.595	7	29	71	0.699
*Pasteurella*	–	–	–	–	2	50	50	> 0.999
*Moraxella catarrhalis*	3	67	33	0.256	3	100	0	0.059
*Candida spp.*	–	–	–	–	2	50	50	> 0.999
*Aspergillus*	–	–	–	–	2	50	50	> 0.999
*Serratia*	–	–	–	–	1	0	100	> 0.999
*Escherichia coli*	1	0	100	> 0.999	–	–	–	–
*Mycobacterium no-TBC*	–	–	–	–	1	100	0	0.396
Polymicrobial	20	35	65	0.848	8	50	50	0.708
Classification according to the conventional treatment^b^: MSCT	31	45	55	**0.026**	51	51	49	**0.014**
MRCT	20	15	85		30	23	77	
MRCT colonisation^c^	–	–	–	–	10	10	90	**0.009**
*Influenza B virus*	–	–	–	–	2	50	50	> 0.999
*Respiratory syncytial virus*	–	–	–	–	7	43	57	> 0.999
*Rhinovirus*	–	–	–	–	7	29	71	0.450
*Parainfluenza virus type 1*	–	–	–	–	3	67	33	0.615
*Parainfluenza virus type 3*	–	–	–	–	3	0	100	0.499
*Parainfluenza virus type 4*	–	–	–	–	1	100	0	0.487

^a^Data reported as percentage related to number of cases for each of sputum sample. ^b^Patients considered MSCT (for aminopenicillin with clavulanic acid, a macrolide or a tetracycline) or MRCT (*Pseudomonas aeruginosa*, MRSA, *Stenotrophomonas maltophilia*, Enterobacteriaceae producer of extended spectrum of beta lactamase and *Acinetobacter baumannii*) [2]. ^c^Colonisation was defined for a positive culture for the same microorganism in the sputum sample collected in the previous year and during hospitalisation. *p* value was calculated versus patients with a negative sample

*Abbreviations: LHS*, length hospital stay; *MRSA*, methicillin-resistant Staphylococcus aureus; *MSCT* and *MRCT*, microorganisms sensible and resistant to conventional treatment

### Study outcomes

**Fig. 3:**
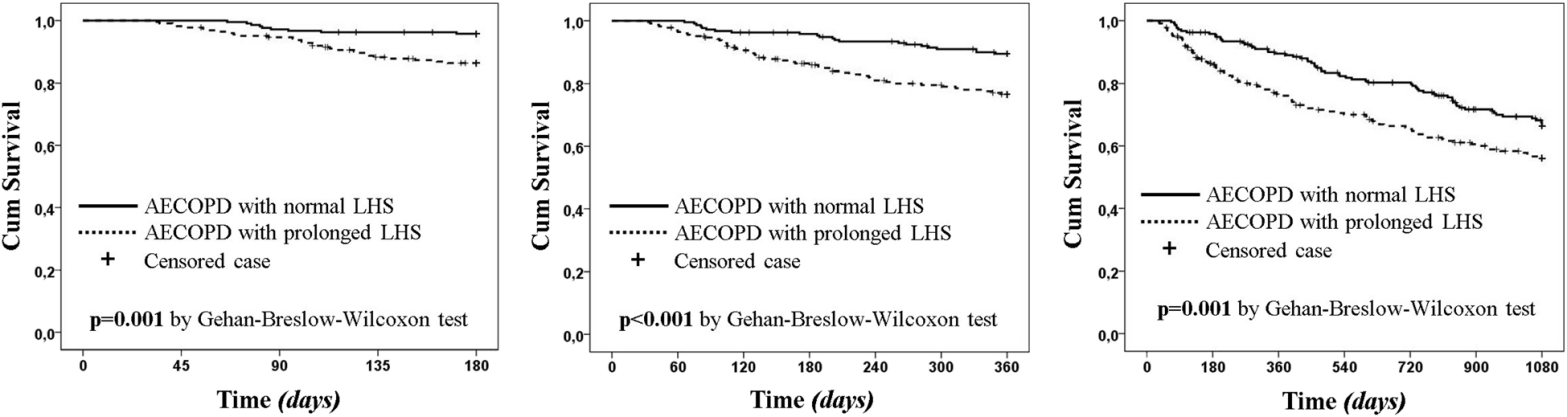
Kaplan-Meier survival curves at 6 months, 1 year and 3 years by length of hospitalisation

All the outcomes (i.e., NIMV use, IMV use, ICU admission, and mortality) were worse in the prolonged LHS group compared with the normal LHS group (Table 4). Similarly, the survival time and the Kaplan–Meier curves in the three follow-up periods (i.e., 6 months, 1 year and 3 years) were significantly different between groups, with worse prognosis for patients with prolonged LHS (Fig. 3).

**Table 4:** Study outcomes

Variables	Normal LHS (≤ 7 days) *(n = 213)*	Prolonged LHS (> 7 days) *(n = 224)*	*p* value
NIMV, %	11	31	**< 0.001**
IMV, %	0	7	**< 0.001**
ICU admission, %	5	19	**< 0.001**
Mortality at 6-months, %	4	14	**< 0.001**
Survival time	176.3 [173.8 to 178.8]	168.5 [164.3 to 172.7]	**0.001**
Cumulative mortality at 1-year, %	11	25	**< 0.001**
Survival time	342.5 [334.4 to 350.5]	313.6 [301.1 to 326.2]	**< 0.001**
Cumulative mortality at 3-years, %	37	48	**0.036**
Survival time	904.6 [862.3 to 946.8]	783.8 [730.6 to 837.1]	**0.007**

Data are shown as number of patients (percentage) and calculated for non-missing data

Survival time was calculated as mean [95% confidence interval] and reported as days

*Abbreviations: NIMV* and *IMV* indicate non-invasive and invasive mechanical ventilation, respectively; *ICU*, intensive care unit

### Prediction analyses

Several clinical variables predicted LHS in the univariate analyses (Table 5). The following were significant predictors of an increased risk of a prolonged LHS: mMRC dyspnoea score ≥ 2, GOLD stage B and D, ≥1 previous AECOPD requiring hospitalisation, LTOT use, symptom duration ≥7 days before admission, drug use before admission (antibiotics three months before and salbutamol and ipratropium two weeks before), COPD-SS questionnaire ≥15, the presence of a congestive heart disease, acute respiratory acidosis or severe hypoxemia at admission, hypercapnia at admission and day 3, renal bicarbonate retention at admission, the need for NIMV, the presence of *P. aeruginosa* or of MRCT colonisation and the use of cephalosporins during hospitalisation. The presence of *S. pneumonia* and the use of fluoroquinolones were associated with a reduced risk of a prolonged LHS.

**Table 5:** Univariate and multivariate regression analyses predicting the probability to have a prolonged hospitalisation

Variable	Univariate			Multivariate		
	OR	95% CI	*p* value	OR	95% CI	p
mMRC dyspnoea score (≥ 2)	2.07	1.29 to 3.32	**0.002**	2.24 (2.76)	1.34 to 3.74 (1.54 to 4.92)	**0.002 (0.001)**
GOLD 2017 stages: Stage A	1.00					
Stage B	2.24	1.22 to 4.08	**0.009**	–	–	–
Stage C	1.48	0.68 to 3.20	0.322	–	–	–
Stage D	2.60	1.35 to 4.98	**0.004**	–	–	–
No. of previous AECOPD requiring hospitalisation (≥ 1)	1.57	1.05 to 2.35	**0.030**	–	–	–
Use of LTOT	1.72	1.12 to 2.64	**0.014**	–	–	–
Onset of symptoms until admission (≥ 7 days)	1.49	0.99 to 2.42	**0.053**	–	–	–
Use of antibiotics three months before admission	1.72	1.01 to 2.93	**0.044**			
Use of salbutamol two weeks before admission	3.44	1.59 to 7.43	**0.002**	–	–	–
Use of ipratropium two weeks before admission	2.84	1.34 to 6.01	**0.006**	–	–	–
COPD-SS (≥ 15 score)	1.39	0.94 to 2.05	**0.095**	–	–	–
Presence of congestive heart disease	2.14	1.21 to 3.79	**0.009**	–	–	–
Acute respiratory acidosis at admission^a^	2.41	1.50 to 3.88	**< 0.001**	2.75 (2.68)	1.49 to 5.05 (1.34 to 5.38)	**0.001 (0.005)**
Hypercapnia at admission^b^	1.90	1.26 to 2.87	**0.002**	–	–	–
Hypercapnia at day 3^b^	2.05	0.97 to 4.32	**0.061**	–	–	–
Acute severe hypoxemia at admission^cd^	2.32	1.14 to 4.73	**0.020**			
Renal bicarbonate retention at admission^e^	1.84	1.21 to 2.80	**0.004**			
Need for NIMV	3.53	2.12 to 5.88	**< 0.001**			
*Pseudomonas aeruginosa* in the sputum sample of the previous year^f^	4.75	0.96 to 23.34	**0.055**			
MRCT colonisation^f^	9.84	1.23 to 78.59	**0.031**			
*Pseudomonas aeruginosa* in the sputum sample during hospitalisation^f^	7.33	2.00 to 26.88	**0.003**			
*Streptococcus pneumoniae* in the sputum sample during hospitalisation^f^	0.33	0.11 to 0.96	**0.042**			
Use of cephalosporins during hospitalisation	4.54	0.98 to 21.06	**0.053**			
Use of fluoroquinolones during hospitalisation	0.61	0.40 to 0.93	**0.023**			

In the univariate model the statistical significance considers a *p* value < 0.1. Data in parentheses report the multivariate model adjusted for anthropometric variables. Hosmer and Lemeshow Test *p* = 0.956 and *p* = 0.642 in the multivariate and multivariate adjusted model, respectively

^a^Acute respiratory acidosis: pH < 7.35; ^b^Hypercapnia: PCO_2_ > 45 mmHg; ^c^Acute severe hypoxemia: PaO_2_/FiO_2_ ratio < 200; ^d^Analysis excluding patients with LTOT; ^e^Renal bicarbonate retention: HCO_3_ > 30 mmol/L; ^f^Definition and criteria are reported in Table 3

*Abbreviations: COPD*, chronic obstructive pulmonary disease; *COPD-SS*, COPD severity score questionnaire; *GOLD*, global initiative for chronic obstructive lung disease; *HCO*_*3*_^−^, serum bicarbonate; *LTOT*, long-term oxygen therapy; *mMRC* indicate modified Medical Research Council; *MRCT*, microorganisms resistant to conventional treatment; *NIMV*, non-invasive mechanical ventilation; *PaCO*_*2*_, partial arterial carbon dioxide pressure; *PaO*_*2*_*/FiO*_*2*_, ratio of partial arterial oxygen pressure to the fraction of inspired oxygen

In the multivariate and multivariate adjusted analyses, an mMRC dyspnoea score ≥ 2 and the presence of acute respiratory acidosis at admission were independently confirmed as predictors of a prolonged LHS.

### Readmissions and LHS

**Fig. 4:**
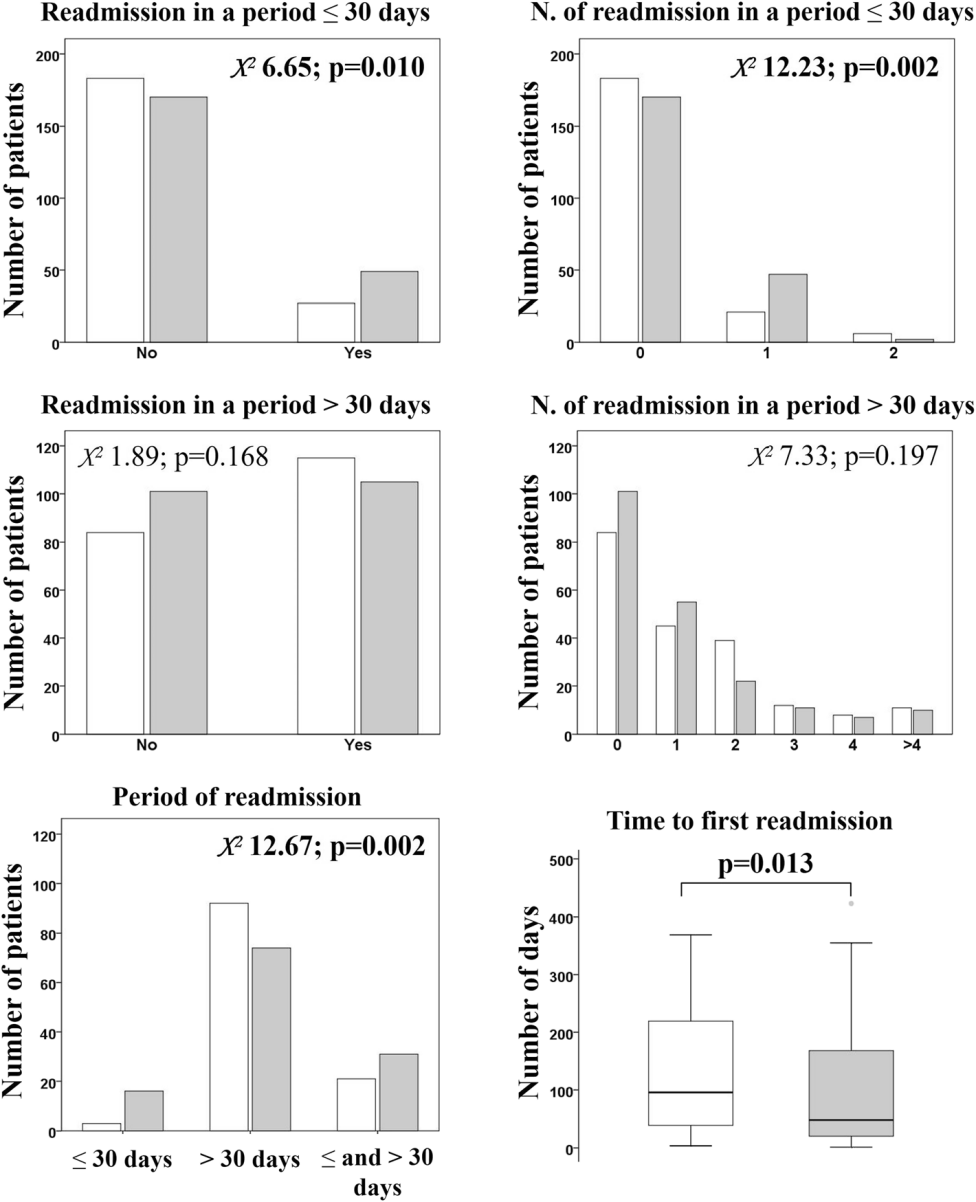
The readmission rates were evaluated for the periods ≤30 days and > 30 days from discharge. White and grey represent the AECOPD patients with normal LHS and prolonged LHS, respectively

In comparison with the normal LHS group, the prolonged LHS group had higher probability of readmission, especially for one readmission and for the period ≤30 days after discharge (Fig. 4). The time to first readmission was lower in the prolonged LHS group compared with the normal LHS group (47 days [19; 167] versus 95 days [38; 221].

### Supplementary analysis not shown

We repeated all predictive and prognosis analyses after excluding patients admitted to ICU during hospitalisation (*n* = 52) or who required IMV (*n* = 17) [16] or who required facilities at discharge (*n* = 25) and the results were equal to those reported for the full cohort. Therefore, we have excluded this detail from the main report.

## Discussion

This prospective observational study of hospitalised patients for AECOPD allows us to make three important conclusions. First, we showed that a LHS of ≥7 days identified a typology of severe AECOPD with common chronic baseline characteristics, including worse disease staging, worse symptom perception, LTOT use and colonisation by *P. aeruginosa* or MRCT. These patients were also at high risk of early readmission to hospital. Second, patients with a prolonged LHS had a worse prognosis until at least 3 years after discharge. Third, at hospital admission, a severe perception of dyspnoea (mMRC scale ≥2) and the presence of acute respiratory acidosis significantly increased the risk of a prolonged LHS. These three aspects of a presentation could help us focusing which patients are at risk of prolonged LHS and ultimately, providing tailored interventions [17–19].

Regarding the threshold for defining a prolonged LHS, we have already noted the existing variations [7–10]. As outlined, we used the threshold of 7 days in line with the time when complications typically develop in an AECOPD [12]. Overall, the baseline characteristics of our AECOPD cohort were comparable to those of other studies [6, 7, 20], but we also considered variables not previously reported in this field. These included the symptom duration before admission and the use of antibiotics and the received therapy in both the short- and the long-term before hospitalisation. These allow us to state that prolonged LHS was related to increased treatment requirements in the period just prior to admission; for example, this group had higher rates of antibiotic use in the 3 months before the admission and higher rates of as-needed bronchodilator use in the 2 months before the admission (Table 1).

Concerning to the microbiological results, our data support those showing a close relationship between bacterial infection and the LHS [21]. Although coinfection with bacteria and viruses may also prolong hospitalisation [21], we did not find a statistically significant association. By contrast, we showed that *P. aeruginosa* [22] and MRCT [23] are clinical factors that influence outcomes and prolong hospitalisation. Consistent with these microbiological findings and data about the impact of treatment failure [24], patients treated with fluoroquinolones during hospitalisation had a lower risk of a prolonged hospitalisation (Table 5). Finally, we believe that it was noteworthy that colonisation with *P. aeruginosa* or MRCT (Tables 3 and 5) played a role in prolonging the LHS.

We showed, for the first time, that patients with a prolonged LHS have a higher risk of mortality until at least 3 years after discharge. This is in line with research that looked at the need for intubation and IMV with regards the LHS [16]. Given this close relationship between ICU admission and prolonged LHS, we repeated all our analyses after excluding patients transferred to ICU, but obtained similar results. The high prevalence of early readmissions (≤ 30 days the discharge) in the prolonged LHS group confirmed the results of previous research [25]. That said, it should be noted that the previous research considered the threshold for a prolonged LHS to be > 4 days, which is probably too short to cure a severe AECOPD. Nevertheless, we contend that a prolonged LHS reflects a severe and chronic underlying disease, resulting in a subgroup with a worse prognosis [26, 27].

### Predictors of long hospital stay

Univariate analysis indicated that several variables predicted a prolonged LHS and this could be useful to develop integrated interventions. However, the multivariate analysis, adjusted for anthropometric characteristics, showed that only dyspnoea perception and acute respiratory acidosis significantly increase the risk of a prolonged LHS. These clinical conditions have both been associated with prolonged LHS [6, 7, 9, 20], though the respective researches used different measures of respiratory acidosis, such as dyspnoea [9, 20], hypercapnia [7] or the need for NIMV [6]. Moreover, these data were not prospectively obtained [6, 7] and they used different study designs [6, 7, 10], methodologies [6, 7, 10] and settings [8, 9].

Only the study of Tsimogianni et al. [10] considered the mMRC and body mass index as independent predictors of a prolonged LHS, but they used retrospective analysis and a threshold of > 8 days to indicate a prolonged LHS [10]. In the same cohort, our results integrate a subjective patient-reported variable (dyspnoea by mMRC) with an objectively measured variable (pH by blood gas analysis), which may provide an opportunity to develop tailored interventions. It is true that the presence of acute respiratory acidosis correlates to the need for NIMV and independently of the need for ICU admission or IMV it will require more time for cure (e.g., adapting to mechanical ventilation and recovery from acute respiratory failure). However, we believe that there is also a need to focus on the patient’s self-report of dyspnoea given its accuracy as a predictor of the LHS. In patients with COPD, the mMRC questionnaire is a simple measure of dyspnoea perception [28, 29] that is adequate for symptom assessment, relating to both health status [30] and exercise tolerance [31]. Moreover, the mMRC has been shown to predict mortality [32]. Our findings confirm the role of mMRC in the prediction of patients with a prolonged LHS.

### Strength and limitation

The main strengths of this observational study were the choice of a definition of prolonged LHS based on clinical experience, the inclusion of many clinical variables, and the long follow-up period to 3 years. However, there were some important limitations. First, although we collected data for more than 400 patients, these derived from a single centre in Spain; therefore, a multicentre study is needed to confirm our results. Second, the research was based on clinical experience by pneumologists and limited to hospital care, so integrations with social variables or economic resources may have been lacking [11]. Finally, related to the potentially effect of in-hospital therapy on LHS, our findings are produced by an observational study and then with difficult comparable. A randomized trial in which all patients receive the same therapy may be useful.

## Conclusions

In conclusion, an mMRC score of ≥2 and the presence of respiratory acidosis at admission predict hospitalisation for > 7 days in patients with AECOPD. Moreover, hospitalisation for > 7 days is a marker of severity that affects major prognostic outcomes. These findings could be clinically relevant by helping to identify at-risk patients when they present to hospital. If these predictors can be shown to be modifiable, which they potentially are, we may be able to offer tailored interventions both before and during admission.

## Abbreviations

AECOPD: Acute exacerbation of chronic obstructive pulmonary disease
BE: Base excess
COPD: Chronic obstructive pulmonary disease
COPD-SS: Chronic obstructive pulmonary disease severity score
CRP: C-Reactive protein
FEV_1_: Forced expiratory volume in the 1st second
HCO_3_^−^: Serum bicarbonate
ICS: Inhaled steroids
ICU: Intensive care unit
IMV: Invasive mechanical ventilation
LHS: Length of hospital stay
LTOT: Long-term oxygen therapy
mMRC: Modified Medical Research Council
NIMV: Non-invasive mechanical ventilation
PaCO_2_: Partial arterial carbon dioxide pressure
PaO_2_/FiO_2_: The ratio of partial arterial oxygen pressure to the fraction of inspired oxygen
